# A new miniaturized extracorporeal membrane oxygenator with integrated rotary blood pump (Ilias): first results in a porcine model of lung injury

**DOI:** 10.1186/cc10703

**Published:** 2012-03-20

**Authors:** K Pilarczyk, J Heckmann, K Lyskawa, A Strauß, U Aschenbrenner, H Jakob, M Kamler, N Pizanis

**Affiliations:** 1 West German Heart Centre Essen, University Hospital, Essen, Germany; 2 iliasmedical GmbH, Bochum, Germany

## Introduction

**Figure 1:**
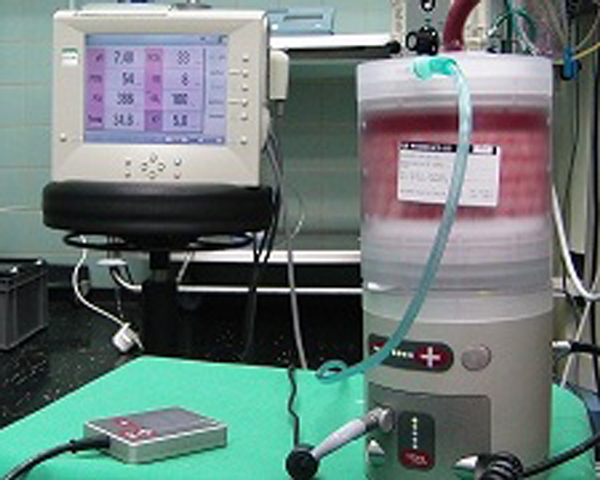
**The Ilias prototype**.

Extracorporeal membrane oxygenation (ECMO) is used for most severe acute respiratory distress syndrome cases in specialized centres. However, critically ill patients fulfilling ECMO criteria are often not suitable for transportation and currently available ECMO systems are not designed for emergency use or interhospital transfer. Therefore, a new miniaturized ECMO (Ilias; Figure 1) with only 5 kg weight was developed to reduce filling volume and simplify management.

## Methods

Acute lung injury was induced with repeated pulmonary saline infusion in 13 pigs until the Horowitz Index was <100 mmHg. Pigs were assigned to the following three groups: group 1 (*n *= 3), control group, conventional ventilation; group 2 (*n *= 5), standard venovenous ECMO (Maquet); group 3 (*n *= 5), Ilias group. Gas exchange, hemodynamics, hemolysis, and coagulation activation were examined over a period of 8 hours.

## Results

No device failed during the observation period. Oxygenation increased significantly in both ECMO groups compared to baseline and to control (paO_2 _from 79 ± 8 before Ilias to 340 ± 108 mmHg and from 61 ± 12 mmHg to 309 ± 59 mmHg in the standard ECMO group). The CO_2 _elimination by the Ilias reduced arterial paCO_2 _from 134 ± 25 mmHg at baseline to 53 ± 7 mmHg. Hemodynamic instability, significant activation of the plasmatic coagulation or platelet consumption was not observed. However, hemolyses were significantly higher in the Ilias group compared to the Maquet group.

## Conclusion

The Ilias prototype provided excellent gas exchange with hemodynamic stability comparable to a standard ECMO system. Further development and design modifications (optimized rotation speed and surface coating of rotor) are already done and another experiment is projected to reduce hemolysis for clinical application.

